# All-Optical Modulation Technology Based on 2D Layered Materials

**DOI:** 10.3390/mi13010092

**Published:** 2022-01-07

**Authors:** Hongyan Yang, Yunzheng Wang, Zian Cheak Tiu, Sin Jin Tan, Libo Yuan, Han Zhang

**Affiliations:** 1 College of Optoelectronic Engineering, Guilin University of Electronic Technology, Guilin 541004, China; hyyang@guet.edu.cn (H.Y.); lbyuan@vip.sina.com (L.Y.); 2 Guangxi Key Laboratory of Automatic Detecting Technology and Instruments, Guilin University of Electronic Technology, Guilin 541004, China; 3 Engineering Product Development, Singapore University of Technology and Design, 8 Somapah Road, Singapore 487372, Singapore; yunzheng_wang@sutd.edu.sg; 4 Faculty of Engineering and Quality Surveying, INTI International University, Persiaran Perdana BBN, Nilai 71800, Malaysia; 5 School of Engineering, UOW Malaysia KDU, Utropolis Glenmarie, Shah Alam 40150, Malaysia; sj.tan@kdu.edu.my; 6 International Collaborative Laboratory of 2D Materials for Optoelectronics Science and Technology of Ministry of Education, Institute of Microscale Optoelectronics, Shenzhen University, Shenzhen 518060, China; Hzhang@szu.edu.cn

**Keywords:** all-optical device, 2D materials, optical modulation

## Abstract

In the advancement of photonics technologies, all-optical systems are highly demanded in ultrafast photonics, signal processing, optical sensing and optical communication systems. All-optical devices are the core elements to realize the next generation of photonics integration system and optical interconnection. Thus, the exploration of new optoelectronics materials that exhibit different optical properties is a highlighted research direction. The emerging two-dimensional (2D) materials such as graphene, black phosphorus (BP), transition metal dichalcogenides (TMDs) and MXene have proved great potential in the evolution of photonics technologies. The optical properties of 2D materials comprising the energy bandgap, third-order nonlinearity, nonlinear absorption and thermo-optics coefficient can be tailored for different optical applications. Over the past decade, the explorations of 2D materials in photonics applications have extended to all-optical modulators, all-optical switches, an all-optical wavelength converter, covering the visible, near-infrared and Terahertz wavelength range. Herein, we review different types of 2D materials, their fabrication processes and optical properties. In addition, we also summarize the recent advances of all-optical modulation based on 2D materials. Finally, we conclude on the perspectives on and challenges of the future development of the 2D material-based all-optical devices.

## 1. Introduction

Moore’s law predicted that the number of transistors in a dense integrated circuit (IC) double every 18 months [1]. As the physical size of IC reaches the fabrication limit, the development of IC is facing constraints to accommodate next generation telecommunication technology, in which smaller sizes of ICs with enhanced functions are in favor. In the past few decades, a lot of resources and efforts have been allocated to overcome the drawbacks of traditional electrical-electronic devices, particularly in intrinsic narrow bandwidth, time delay and Ohmic loss contributed by copper cable interconnections. Hence, photonics and optoelectronics are highlighted as potential solutions to overcome the above challenges and to drive future technologies.

The traditional electrical-electronics devices slowly evolved to silicon-based photo-electrons interconnection technology. It is widely used to bridge the photonics system and electronics systems, in which photonics functionality and electronics intelligence are incorporated together in an integrated design. Such photonics devices tapping on the mature CMOS technology are compact in size, cost effective, have low energy consumption and offer better data processing performance [2]. Substantial development and progress can be seen over the past few decades for silicon-based on-chip optical interconnects, particularly on silicon-based optical modulators, as they are widely used in high level data processing. Nevertheless, the performance of a silicon-based photonic modulator is inherently restricted due to the intrinsic properties of silicon. For instance, the low photoelectric coefficient, thermal instability and carrier mobility of pure silicon materials hinder silicon-based optical modulators to achieve high modulation rate.

To solve the above issues, it is crucial to search for new materials that can be integrated into the silicon substrate to attain a higher modulation performance and lower energy consumption on smaller device size. Active control of light at the nanoscale, enhancement of light–matter interaction, high nonlinear materials and ingenuity component designs play important roles; in particular, material selection and structure design are the requirements that must be taken into consideration to achieve the desired optical modulation performance on a nanometer scale. Contrarily to the typical electronics signal processing and modulation, optical signal processing and modulation heavily rely on nonlinear optical properties in materials.

The past decade had witnessed explosive research in graphene and other 2D materials. In 2004, Novoselov and Geim et al. successfully exfoliated monolayer graphene from graphite using scotch tape, which led to exploration on single atom layer two-dimensional (2D) materials [3,4]. The study in exceptional electronic and photonic properties of 2D layered materials has expanded from graphene to transition metal dichalcogenides (TMDs), topological insulators (TIs), black phosphorus (BP), antimonene, silicene and MXenes [5,6,7,8]. These new 2D layered materials pose many unique optical and electronic properties. For instance, 2D materials exhibit high anisotropy in Young’s modulus and quantum confinement effect. They have rich electronic properties from wideband insulator to narrow-gap semiconductor and semimetal; their properties can be easily modified using doping techniques or by applying potential energy into them. In addition, 2D materials can be easily transferred to an arbitrary substrate without lattice matching requirements. The application of 2D materials was first established as pulsed lasers [9,10,11,12,13,14,15,16,17] and with the introduction of innovative integration techniques, it accelerates the device on-chip applications in optical communication, optical modulation, optical detection as well as optical sensing [18,19,20]. Lately, the venture of 2D materials in the heterostructure, plasma structure, and fiber integrated structure are bringing great feasibility of 2D materials in all-optical devices and next-generation optical communication systems.

In recent years, many review papers have been published to summarize the advances in 2D materials from different perspectives. In 2012, Bao et al. reviewed graphene-based photonics, plasmonics and broadband optoelectronic devices, focusing on the polarizer, modulator and photodetector [21]. The graphene-based ultrafast mode-locked lasers and optical modulators were reviewed by Sun et al. [11,22]. In addition, graphene-based optical modulators using single layer [23] and double layer [24,25] have also been discussed by Xiang Zhang research group. Xia et al. have also intensively discussed the application of 2D materials for future nano-photonics [26]. Furthermore, Luo et al. have extended the discussion, encompassing a graphene-based optical modulator, its physical mechanism and graphene electro-optic modulation from different perspectives of modulation structure and functionality [27]. The advantages of 2D materials in ultrafast all-optical switching has also been discussed by Hu et al. [28]. Tong et al. further detailed the potential of 2D materials in electro-optic, thermo-optic and all-optical modulation [29]. Successively, Song et al. have reported the progress of optoelectronic devices using black phosphorus-based materials, which covered the synthesis and preparation technology of black phosphorus (BP) as well as its potential applications in nanoscale electronics, ultra-fast photonics, nanophotonics and optoelectronics [30,31,32,33].

Early experimental work revealed that modulation speed in the range of 1 GHz to 30 GHz were reported by Liu et al. [23] and Phare et al. [34], including the integration of graphene on silicon and silicon nitride waveguide. Undoubtedly, the discovery of graphene revealed a significant breakthrough in the silicon-based optical modulator, which can overcome the weak modulation capability of silicon and is a promising solution for future optical interconnection. This GHz speed looks promising for 5G requirements and applications. In the era of Internet of Things (IoT) and future 6G wireless communication, more smart devices will be connected, and it is projected that data speed will go beyond 100 GHz. As such, the all-optical network, from fronthaul to backhaul, is projected to achieve super high-speed signal processing. To achieve an all-optical system, light-control-light with the incorporation of 2D materials is one of the focuses in photonics technology. The light modulation can be achieved in the form of amplitude, frequency, phase and polarization of the light. Through the interaction of pump light with 2D materials, the properties of 2D materials can be modified in terms of nonlinearity and refractive index. Successively, the characteristic of signal light will be modulated accordingly at the modulator’s output.

In this paper, we mainly focused on reviewing the advancement of both the fundamental concept and application of all-optical technology using 2D materials, particularly in optical modulation. We are placing the emphasis on optical modulation, as it is one of the main functional components for any optical interconnect solution. The review is organized into three parts. First, we begin to explore different types of 2D materials, which include graphene, TIs, TMDs and MXenes, along with their fabrication process. This is followed by the working principle and types of optical modulators. The types of modulation that we will examine are amplitude modulation, polarization modulation, wavelength modulation and temporal modulation. In the last part of this review paper, we conclude our review by proposing some suggestions on the prospect of future research directions of all-optical modulators using 2D materials.

## 2. Two-Dimensional Materials

**Figure 1:**
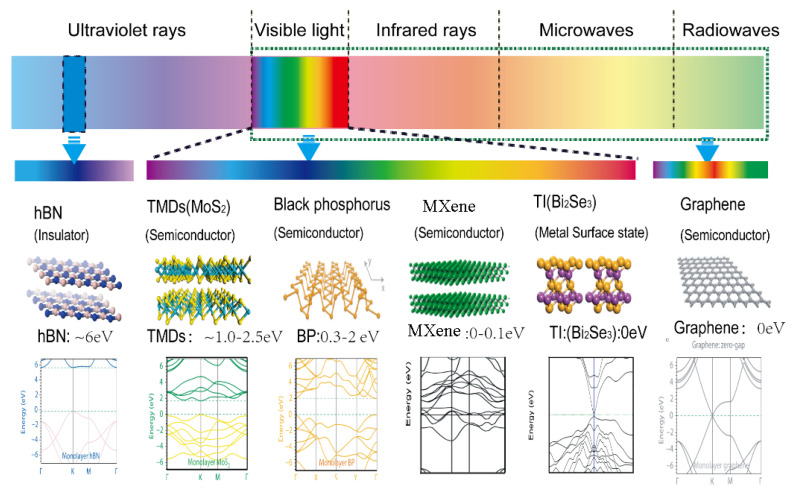
Typical two-dimensional materials: Spectral properties, atomic structures and band diagrams of hBN, TMD, BP, Mxene, TI and graphene.

Since the discovery of carbon nanotube [35] and graphene [36], low-dimensional materials have attracted enormous research interests due to their great potential in next-generation nano-optoelectronic devices. Subsequently, a different type of 2D materials, such as transition metal dichalcogenides (TMD), black phosphorus (BP), and MXene have been explored and widely investigated. Materials which are 2D exhibit strong intra-layer bonding and weak inter-layer bonding, which enable them to form heterostructures without the conventional “lattice mismatch” issue. Furthermore, 2D materials offer great flexibility to be integrated into different photonics structure, including optical fiber and silicon substrate. This allows 2D materials to act as one of the most suitable candidates to combine with other functional materials and to be applied in a wide range of electronics and photonics devices. Figure 1 shows an overview of the atomic structure, operating wavelengths, molecular structure, and band gap diagram of typical 2D materials. We will describe in detail the electrical and optical characteristics of graphene, TI, MXene, BP and TMD in the remainder of Section 2.

### 2.1. Types of 2D Materials and Their Characteristics

#### 2.1.1. Graphene

**Figure 2:**
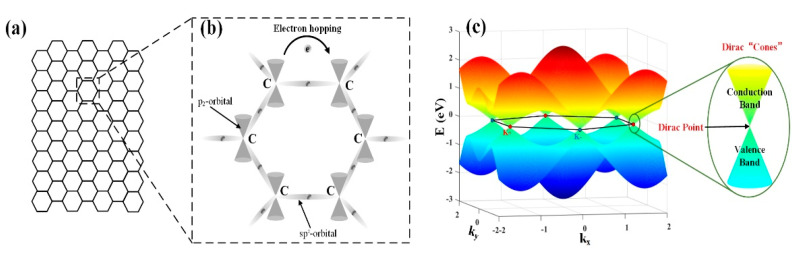
Graphene. (**a**) Monolayer carbon atoms arranged in a honey-comb lattice, (**b**) Electronic hopping and (**c**) The six contact points of the conduction band and valence band are all Dirac points on the Fermi surface. Ref. [1]. Copyright 2014, Elsevier Inc.

Graphene is a 2D allotrope of carbon with atoms arranged in a 2D hexagonal lattice. It has many superior physics and chemical properties and is known as a “dream material”; it is also a promising replacement for silicon on-chip integration technology [37,38]. As shown in Figure 2, graphene is a monolayer of carbon atoms arranged in a honeycomb lattice, which has attracted great interest due to its unique mechanical, and thermal, electronic and optical properties [39,40,41,42,43]. The excellent material properties of graphene have been investigated thoroughly and obtained some remarkable outcomes. For instance, few atomic graphene layers are able to provide superior properties such as being 100 times stronger than steel, as flexible as rubber, tougher than diamond, and 13 times more conductive than copper [44,45]. Graphene is a zero-gap semiconductor that exhibits a novel electronic structure.

Electrical properties of graphene in its special electronic band structure, as shown in Figure 2. There are six points of the conduction band and valence band, which are in the Fermi–Dirac point. Below the contact point position, the electron density of the states is zero. Thus, it is a zero-bandgap structure of a semiconductor and no metal Fermi surface. In addition, monolayer graphene is almost transparent, and exhibits unique optical properties in a wide spectral range. The unusual low-energy electronic structure of graphene is due to the holes and tapered bands of electrons meeting at Dirac point. Further, Dirac fermions with forbidden backscattering allow graphene to exhibit the highest carrier mobility at room temperature. Graphene presents an exceptionally high carrier mobility exceeding 10^6^ cm^2^V^−1^s^−1^ at 2 K and 2 × 10^5^ cm^2^V^−1^s^−1^ at room temperature [46]. As for the optical properties, a strong and universal absorption is brought by the linear dispersion of electrons. It is worth mentioning that graphene is almost transparent, with the optical absorption as low as 2.3% across a broad wavelength range, which covers the infrared and visible range. Owing to the gapless and semi-metallic properties, graphene can interact with light in an extremely wide spectral band from microwave to ultraviolet. Hence, graphene is highly recommended in various photonics and optoelectronics applications [28,47,48,49,50,51,52,53,54,55,56,57,58,59]. Moreover, the broadband property is also favorable to support the optical devices requiring broadband light-matter interactions, such as photodetectors, optical modulators, electromagnetic wave shielding, notch filter, and linear polarizer. Furthermore, graphene is also an excellent platform for plasmonic devices, owing to the active control of its conductivity. The conductivity of graphene can be modified using chemical doping, electric field, or magnetic fields based on the Kubo formula [60].

(1) $$\delta \left(\omega \mu_{c}\Gamma T\right)=\sigma_{intra}\left(\omega ,\Gamma T\right)+\sigma_{inter}\left(\omega ,\Gamma T\right)\;=-\frac{ie^{2}}{\pi \hbar \left(\omega +i2\Gamma \right)}[\int_{0}^{\infty }\varepsilon \left(\frac{\partial f_{d}\left(\varepsilon \right)}{\partial \varepsilon }-\frac{\partial f_{d}\left(-\varepsilon \right)}{\partial \varepsilon }\right)d\varepsilon -\frac{ie^{2}\left(\omega +i2\Gamma \right)}{\pi \hbar }[\int_{0}^{\infty }\frac{f_{d}\left(-\varepsilon \right)-f_{d}\left(\varepsilon \right)}{{\left(\omega +i2\Gamma \right)}^{2}}-4\left(\frac{\varepsilon }{\hbar }\right)d\varepsilon ]$$

where *γ* represents the scattering rate, *µ*_c_ represents the chemical potential, *f_d_*(*ε*) is the Fermi–Dirac distribution, *i* is the imaginary unit, *e* is the charge of the electron, *ħ* is the reduced Planck’s constant, *ω* is the radian frequency, and *T* is the temperature. The chemical potential of graphene, *µ*_c_ (also Fermi level *E*_F_), depends on the carrier density, which can be controlled by external gate voltage, electric field, magnetic field, and chemical doping [21]. With the unique Dirac cone, the properties of graphene can be converted from insulators to metal-like properties. The tunability of the interband transitions in graphene offers new possibilities for in depth study on the fundamental physics for various technological applications. For instance, the incorporation of graphene into the silicon substrate and optical fiber to form an optical modulator resulted in high modulation speed, broad modulation wavelength range, reduction of device footprint as well low energy consumption. More details are discussed in Section 3.

In contrast to the conventional semiconductor materials, where the absorption is limited by their bandgap, graphene absorbs light across a broad electromagnetic spectral range from the ultraviolet to the terahertz. This enables light modulation with a much broader operation wavelength range. As a result, light modulation with graphene has been proven in visible [52,61,62], infrared [23,62,63,64] and terahertz ranges [18,50,65,66,67,68,69,70,71].

#### 2.1.2. Topological Insulators (TIs)

**Figure 3:**
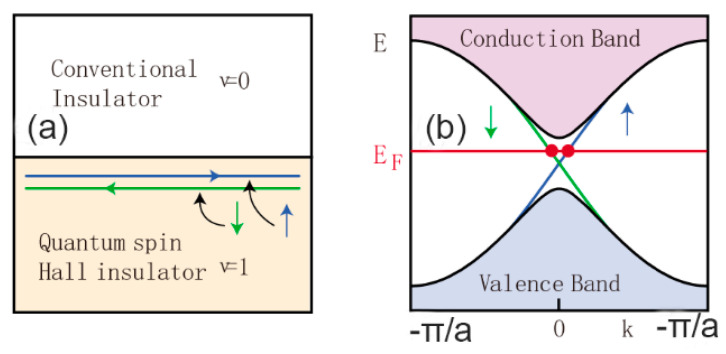
Edge states in the quantum spin Hall insulator (QSHI). (**a**) The interface between a QSHI and an ordinary insulator. (**b**) The edge state dispersion in the graphene model in which up and down spins propagate in opposite directions [50]. Copyright 2012, American Physical Society.

TI is a 2D photoelectric material with internal insulation and a conductive surface or edge. The unique characteristic of this kind of materials is that the strong spin–orbit coupling, which makes it possible to reverse the energy band. TI is the only model exploiting spin–orbit interactions, which is generally related to introducing a time-reversal-symmetry–breaking interaction. TI has stable low-latitude metal states protected by topological protection at the boundary, as shown in Figure 3. The edges of these gaps are excited in the forbidden band and connected to the valence and conductive bands. The red line represents the Fermi surface and the green line represents the edge state. It includes two effects, one is the quantum Hall effect, which is a high magnetic field and a broken time-reversal symmetry [72,73]. The other is time-reversal-invariant topological insulators, which is a highlighted research area in condensed matter physics. TI is insulating in the bulk and conducts along the helical edge states. It was theoretically predicted [74] and experimentally observed [75] in HgTe/CdTe quantum wells. In general, TI can be divided into two categories: one is the quantum Hall system that destroys time inversion, and the other is TI that remains unchanged through time inversion by the recently discovered method. Since 2004, dozens of TIs have been discovered: the first generation is represented by HgTe quantum well; the second generation is represented by compounds such as Bi_2_Se_3_, Bi_2_Te_3_ and Sb_2_Te_3_.

#### 2.1.3. MXenes

MXenes, a new member of the two-dimensional material family, combine early transition metal carbides and carbonitrides. They are produced by selective etching of the A element from the MAX phases, which are metallically conductive, layered solids connected by strong metallic, ionic, and covalent bonds, such as Ti_2_AlC, Ti_3_AlC_2_, and Ti_4_AlC_3_. MXenes combine the metallic conductivity of transition metal carbides with the hydrophilic nature of their hydroxyl or oxygen terminated surfaces. In short, MXenes behave as “conductive clays”. As a result, MXenes are believed to be suitable candidates for energy related applications. For instance, MXenes have shown promising performance in electrochemical energy storage system [76]. All the MXenes are metallic in the absence of surface functionalization. Therefore, some MXenes behave as semiconductors with energy gaps about 0.25 to 2.0 eV [77]. Due to its unique two-dimensional layered structure, large ratio surface area, good conductivity, chemical grafting functional groups and other characteristics, it can be widely used in energy storage, catalysis, adsorption, hydrogen storage, sensors, and new polymer-reinforced matrix composite materials and other fields [78,79]. For example, MXene’s research on energy storage focuses on lithium-ion batteries [80,81,82,83], supercapacitors [84,85,86,87,88], and fuel cells [89,90,91]. The typical two-dimensional layered structure of MXene, with a large ratio surface area, can absorb heavy metals, harmful anions, and organic pollutants, which can effectively purify drinking water and is expected to be used in air and sewage purification [92,93]. In addition, MXene has excellent friction reduction performance and can be used as lubricating oil [94,95] and additive for base oil.

#### 2.1.4. Transition Metal Dichalcogenides (TMDs)

**Figure 4:**
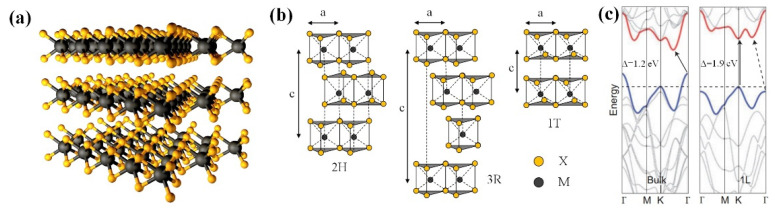
TMDs materials (**a**) MX_2_ structure, with the chalcogen atoms (X) in yellow and the metal atoms (M) in grey, (**b**) Structural polytypes: 2H, 3R and 1T, (**c**) The energy band for different materials.

TMD is a 2D material with MX_2_ structure, in which M is transition metal and X is chalcogen. The structural polytypes is shown in Figure 4: 2H (hexagonal symmetry, two layers per repeat unit and trigonal prismatic coordination), 3R (rhombohedral symmetry, three layers per repeat unit and trigonal prismatic coordination) and 1T (tetragonal symmetry, one layer per repeat unit and octahedral coordination). The lattice constants of TMDs are in the range between 3.1 to 3.7 Å. In addition, the stacking index c indicates the number of layers in each stacking order, and the interlayer spacing of TMDs is around 6.5 Å [96].

TMDs are layered materials with strong intralayer bonding and weak interlayer bonding. This property enables exfoliation into two-dimensional layers with single unit cell thickness. Two-dimensional TMDs are MX_2_ compounds, where M is a transition element from groups IV, V and VI of the periodic table, and X represents the chalcogen, which includes S, Se, and Te. The band structure of TMDs highly relies on their number of layers [97]. TMDs exhibit unique electrical and optical properties that evolve from the quantum confinement and surface effects. Moreover, TMD material is able to transform from indirect bandgap to a direct bandgap when bulk materials are scaled down to monolayers. For instance, bulk MoS_2_ is an indirect band semiconductor, whereas monolayer MoS_2_ is a direct band material [98]. The layer dependence properties allow TMD to cater to the different application by manipulating TMDs in nanoscale. Different from the zero-gap band structure of graphene, 2D TMD nanomaterials offer a wide range of electronic properties from an insulator to metal. MoS_2_, MoSe_2,_ and WS_2_ exhibit semiconducting behaviors, which are feasible to be used in electronic devices, whereas NbSe_2_ is metallic and exhibits superconductivity at low temperatures. TMDs are almost as thin, transparent and flexible as graphene. Moreover, TMDs pose a semiconductor nature, which is favorable to integrate with ultra-small and low power transistors and cope with ever-shrinking devices [97]. Few-layer TMDs exhibit a variety of electronic band structures, covering conductor, semi-metal, semiconductor, insulator and superconductor materials. In addition, the change of TMD layer enables the alteration of the energy band gap within 1~2.5 eV, and modifies the indirect band gap into a direct band gap, giving it a wide range of spectral characteristics. Characteristics and properties of TMD in comparison with other materials can be referred to Table 1.

**Figure 5:**
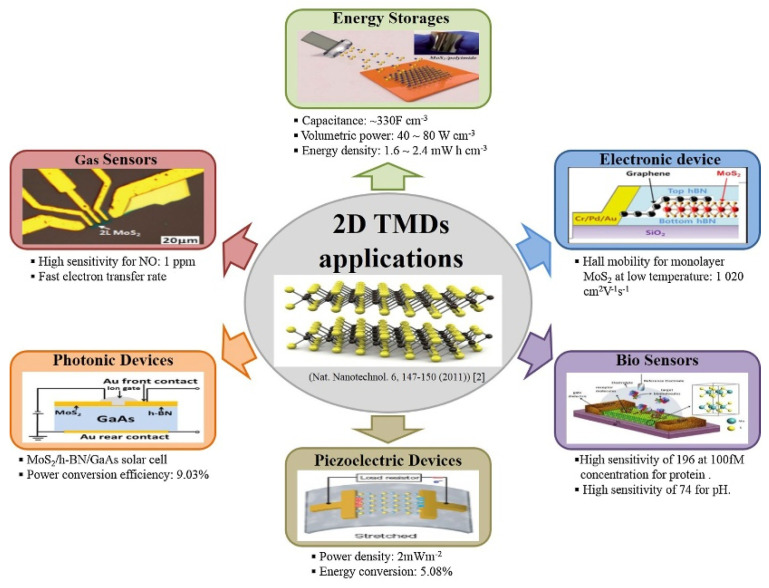
Typical applications of 2D TMDs. Reprinted with permission [2,3,4,5,6,7]. Ref. [2] Copyright © 2015, The Author(s); Ref. [3] copyright © 2014, Nature Publishing Group, Ref. [4] Copyright © 2015 The Royal Society of Chemistry, Ref. [5] Copyright © 2014 American Chemical Society, Ref. [7] Copyright © 2015, Nature Publishing Group.

Therefore, TMDs can overcome the disadvantage of graphene (zero bandgaps) and maintain the advantages of flexibility as well as atomic thickness. As a result, TMD materials are highly potential to be a semiconductor material that can replace silicon. With appropriate dimensions, thickness, flatness, flexibility, bandgap, and high carrier mobility, TMDs can form vertical heterojunctions, planar heterojunctions, and superlattice junctions, which are highly sought after in the development of a new generation of electronic and optoelectronic devices. On the other hand, TMDs have similarities with ubiquitous silicon of a bandgap in the visible-near IR range, high carrier mobility, and a large on/off ratio. Thus, TMDs are more suitable to deposited onto flexible substrates to provide stress and strain compliance as compared to silicon. To date, there are many researchers are exploring TMDs in a wide range of applications such as transistors, photo-switches, supercapacitors, additives for mechanical reinforcement, gas sensors and electrochemical catalysis, as elaborated in Figure 5.

#### 2.1.5. Black Phosphorous (BP)

There are many potential 2D semiconductor materials. However, 2D semiconductor materials that can realize the continuous regulation of single-component band gap materials are limited. Among the 2D semiconductor materials, BP exhibits a honeycomb folded layer structure, which is suitable to function as a laminated semiconductor material with an adjustable bandgap and can compensate for the energy gap between the zero-bandgap graphene and the relatively large-bandgap of TMDs. BP exhibits 0.3 eV in bulk and can be expanded to 1.0–1.5 eV depending on the number of layers. The range of phosphorene band gap corresponds to an absorption spectrum between visible light to infrared. Moreover, the band gap of phosphorene is also highly sensitive to the strain either in-plane or out-of-plane. In the electronics application, phosphorene-based field effect transistor (FET) exhibits high mobility and high on/off ratios. Unlike other 2D materials, phosphorene has in-plane anisotropy, which is suitable for polarized light detection. Hence, black phosphorus with an adjustable bandgap and high carrier mobility (as shown in Figure 1), is a promising material in the field of photoelectricity and optical detection and exhibits promising properties for near- and mid-infrared photonics.

In this section, we have discussed the typical 2D material characteristics. In general, 2D materials have the following characteristics: low mass, high mobility and excellent photoelectric properties. The review in this section has also expanded to 2D energy band characteristics and the related field effects of its large specific surface area and flexibility. Owing to the integration flexibility, excellent optical and electrical properties, the emergence of 2D materials are creating a new development platform for the next generation photonic integration and all-optical devices.

### 2.2. Fabrication of 2D Materials

**Figure 6:**
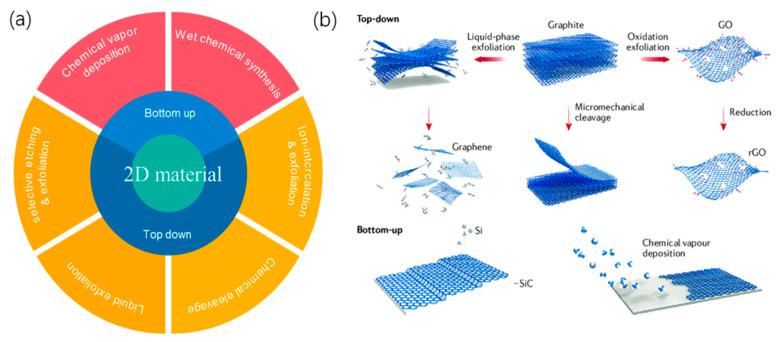
Overview on bottom-up and top-down approach on 2D material fabrication. (**a**) Classification of fabrication methods of 2D materials; (**b**) Schematic diagram of these two fabrication methods using graphene as an example.

There are two main methods for the preparation of 2D layered materials [99]: (a) the top-down method, by breaking the van der Waals force between the layers, which dissolves the bulk material into a single layer or few layers of 2D nano-sheets; and (b) The bottom-up approach is to synthesize two-dimensional materials directly from the molecular level. Figure 6 shows an overview of top-down and bottom-up fabrication methods from the macroscopic view.

#### 2.2.1. Top-Down Approaches (Exfoliation from Bulk)

In the top-down approach, different forces are applied to exfoliate the layered bulk structure to a single layer or few-layer 2D structure. The top-down method involves physical methods such as mechanical force, ultrasonic wave, and thermal stress to destroy the van der Waals force between the 2D layers of material. In these layered crystals, intra-planar covalent bonding is very strong but the inter-planar van der Waals force is comparatively very fragile. Thus, in all the top-down methods, various external forces are exerted to break this weak van der Waals force in between the planes in the layered bulk compounds. The advantages of the top-down method are raw material availability, simple operation, and generally not needing a high temperature or high technology equipment. However, these techniques are not promising to control the number of layers, nor to achieve a uniform and large area of 2D materials flake. Among the top-down methods, the scotch-tape (mechanical exfoliation) technique is the simplest and lowest-cost technique that does not involve any chemical equipment. Liquid phase exfoliation (LPE) technique is one of the most common techniques to exfoliate a few layers of 2D materials from the bulk structure. In this technique, bulk material is mixed with suitable solvents. Typically, N-Methyl pyrrolidone (NMP) and dimethyl formamide (DMF) are used as solvents for most of the 2D materials. Subsequently, the mixture will undergo a sonication process. The sonification process will break the inter-planar van de Walls bonding, but it is not strong enough to break the intra-planar covalent bonding. LPE has been widely used to develop a different type of 2D ultrathin nanosheets [87,88,89,90] due to the satisfactory yield rate and the lateral size of the 2D materials sheets being reasonably large.

Among the top-down methods, the ion-intercalation and exfoliation methods are less popular. In ion-intercalation and exfoliation methods, ions are intercalated into the interplanar spacing of the layered bulk materials by the weakening of the van der Waals force under sonication. Generally, metal ions are actually intercalated into the interlayer structure of the bulk material. From the literature, Hua Zhang et al. developed an electrochemical method to produce Li+ ion-intercalated layered compounds [91,92,93]. This method can greatly enhance the production yield as compared to the aforementioned. However, this method required high temperatures and a long period of processing time. Moreover, intercalated organometallic compounds are highly explosive when they come into contact with air or oxygen. As a result, the feasibility of this technique is constrained due to its shortcoming.

#### 2.2.2. Bottom-Up Approach

In the bottom-up approach, nanomaterials are assembled from basic building blocks, such as molecules or nanoclusters. Chemical vapor deposition (CVD) uses gaseous materials on the surface of solid chemical reactions to generate a solid sediments process. This is a method to obtain solid materials by putting reactants in the gaseous environment, forming solid objects through chemical reactions and depositing them on the surface of the heated solid matrix [3]. This is a promising technique for forming a functional film on a substrate by layering single or multiple films on the surface of a variety of materials or products, using the reaction between the gas phases, thereby enabling the materials or products to obtain various excellent properties required. The nanostructures are synthesized onto the substrate by stacking atoms onto each other, which gives rise to crystal planes. Successively, crystal planes further stack onto each other, resulting in the synthesis of the nanostructures. A bottom-up approach can thus be viewed as a synthesis approach, in which the building blocks are added onto the substrate to form the nanostructures. Taking graphene as an example, the method is to grow graphene on a substrate by breaking the chemical bonds of carbon compounds by means of heating and electron bombardment. Based on raw materials and carbon atom sources, bottom-up synthesis method can be further subdivided into heating SiC method, carbon metal degradation method, chemical vapor deposition (CVD) method, molecular epitaxial growth method and chemical synthesis method (oxidation graphite reduction method).

## 3. Working Principles and Types of Optical Modulator

An optical modulator is a device that is used to vary the fundamental characteristics of light. It could be a change of amplitude, frequency, temporal phase or polarization of light in free space or optical waveguide. The change of light characteristics can be achieved by changing the refractive index ($\hat{n}=n+ik)$ f material that builds up in the optical modulator. The refractive index can be changed by injecting external stimuli such as temperature, pressure, magnetic field, optical field and electric field into the material. Traditional optical modulators are using group IV and III-V (GaAs) and silicon (Si) semiconductors. However, limited spectral range and modulation speed due to their energy band and carrier transit time make it difficult to achieve UWB (ultra-wide bandwidth) optical modulation, and it is not suitable for some applications that require more stringent device performance. The unique photoelectric properties and high third-order nonlinear characteristics of two-dimensional materials are offering a great opportunity to overcome the drawback faced by traditional bulk materials. Materials which are 2D reveal many fascinating properties that can offer better performance for optical modulation. For instance, 2D materials are an atomically thin material, demonstrating strong optical nonlinearities, including harmonics generation, four-wave mixing, Kerr effect, and other nonlinear effects [100]. The quantum confinement in the direction perpendicular to the 2D plane leads to novel electronic and optical properties that are distinctively different from their bulk parental materials [26]. Moreover, 2D materials have also shown better mechanical flexibility, robustness, ease of fabrication and integration. As a result, 2D materials have been used in many types of photonic integrated circuits. The applications of 2D materials have also expanded to the multifunction integrated photonic and electronic circuits [22]. Undoubtedly, the unique optical properties of 2D materials have evolved in the field of nano-photonics, particularly in the development of an all-optical modulator. In light-control-light operation, the carrier densities and distribution in 2D materials change when excited with an external light pulse. This induces a variation in the real and imaginary refractive index of 2D materials. Here, we will review different types of optical modulation (change of light characteristics) based on their working principles: (i) saturable absorption and Kerr effect, which is related to the third order nonlinear response of a material and also (ii) termo-optic effect.

### 3.1. Saturable Absorption

Saturable absorption refers to the phenomenon of the light absorption of the material decreases with the increase in incident light. The imaginary part of refractive index accounts for optical saturable absorption [101], a property of materials in which the absorption coefficient decreases as incident light intensity increases and becomes saturated at a steady level with sufficiently high intensity. A material with such a characteristic is regarded as a saturable absorber (SA). SA exhibits an intensity-dependent transmission, in which the absorption of light decreases to the saturated level at a sufficiently high incident light intensity [21].

The key performances for a SA are its operating wavelength, recovery time, dynamic response, modulation depth, and saturation intensity. Most of the 2D materials exhibit absorption coefficient (*α*) decreases with the increase in high intensity laser. The dependence of the measured absorption coefficient *α* on the intensity *I* of the incident laser radiation is given by the expression [3]:

(2) $$\alpha =\frac{\alpha_{0}}{1+I/I_{s}}$$

where $\alpha_{0}$ is the low-intensity absorption coefficient, and *I*_s_ is known as saturation intensity. The transmittance *T* has a relation with the input intensity *I* which is denoted as:

(3) $$T=1-\frac{\alpha_{s}}{1+I/I_{s}}-\alpha_{ns}$$

where $\alpha_{s}$ is the saturable absorption component, also termed as the modulation depth, $\alpha_{ns}$ is the non-saturable absorption component, *I*_s_ is the saturable intensity, defined as the optical intensity when the optical absorbance is reduced to half of its unbleached value. When an incident light is incident on 2D material, its valence band electron transition becomes depleted, and the conduction band will be occupied [102]. During the carrier relaxation process, absorption of 2D material will reduce with the increase in the input intensity [103]. This resulted in the saturation absorption effect, which is accountable for inducing the temporal modulation, by changing a continuous wave (CW) into a pulsing operation, namely Q-switched and mode-locked pulsing [9,11,12,14,16,104,105,106,107,108,109,110,111].

**Figure 7:**
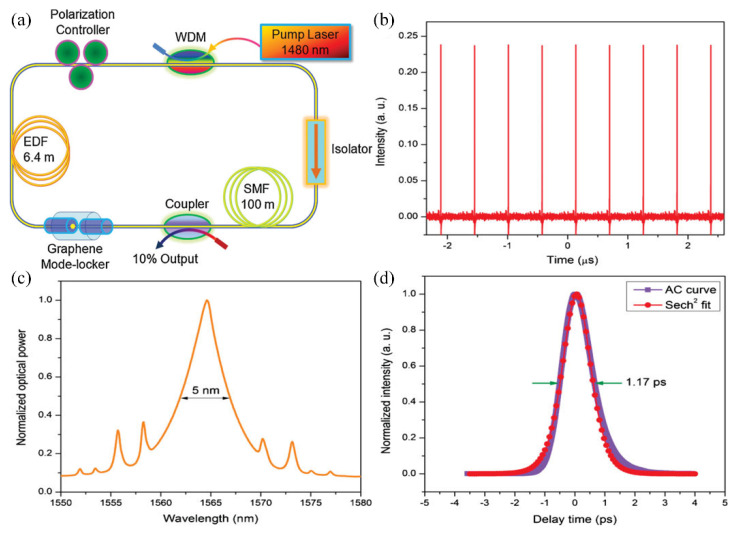
CVD-grown graphene mode-locked EDFL. (**a**) Laser configuration; (**b**) output pulse train; (**c**) output laser spectrum; (**d**) autocorrelation trace. [112]. Reproduced with permission, Copyright 2009, Wiley-VCH.

The typical set-up for pulsing operation is as shown in Figure 7. In 2009, Bao et al. reported the first passive temporal modulation, employing a graphene erbium doped fiber laser (EDFL) [112]. He reported temporal mode-locked pulsing with a repetition rate of 1.79 MHz and a pulse width of 756 fs with a CVD grown graphene film sandwiched between fiber ferrule. Luo et al. demonstrated mode-locked pulsing with BP coated taper fiber, resulting in 4.96 MHz repetition rate and pulse width of 940 fs [113].

An area of interest in mode-locked pulsing is the change of pulse width with pump power, which is regarded as dissipative soliton resonance. In dissipative soliton resonance, the temporal pulse width can be varied while its repetition rate is maintained. Lee et al. discovered the formation of such a pulse by using Bi_2_Te_3_ topological insulator, in which the temporal pulse width was observed to change from 2.7 ns to 12.8 ns, with the increment of pump power [114]. Another interesting work demonstrated on dissipative soliton resonance was shared by Cheng et al. [115]. Pulse width was observed to change broadly, from 0.52 ns to 60.8 ns, while the peak amplitude remained constant. He utilized graphene oxide, one of graphene derivatives, as an SA to operate in 1 μm region. He concluded that the generation of dissipative soliton resonance pulse with clamped peak amplitude was due to reverse saturable absorption.

**Figure 8:**
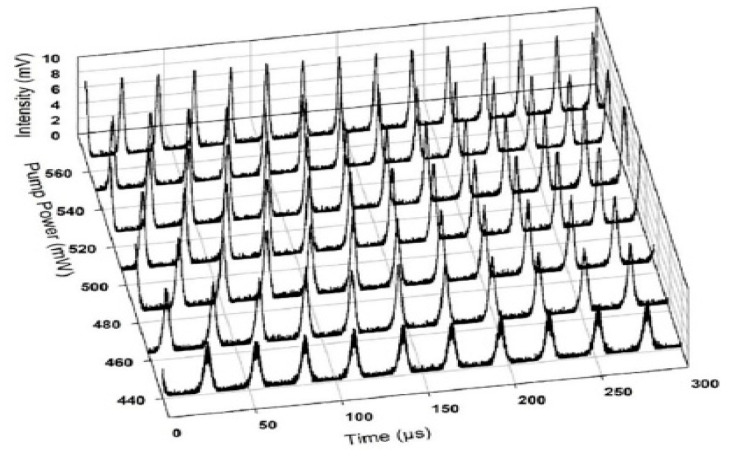
Evolution of the repetition rate of Q-switched laser.

Temporal modulation can also be demonstrated via Q-switching pulsing operation using SA. Contrary to mode-locking, the repetition rate of Q-switching changes with pump power. Many works were demonstrated on Q-switching. Figure 8 shows the evolvement of repetition rate for a typical Q-switched pulse, in the sub kilo Hertz range, ranging from 10 to 200 kHz. Popa et al. used graphene, with repetition rate ranging from 36 kHz to 103 kHz [116]. Li et al. used WS_2_ to obtain Q-switched pulse with repetition rate ranging from 15 kHz to 62 kHz [117]. Q-switching with Bi_2_Se_3_ was demonstrated by Haris with tunable repetition rate from 14.96 kHz to 62.5 kHz in 1550 nm region and 23 kHz to 36 kHz in the 1 μm region wavelength [118]. The properties of SA play an important role in determining the formation of mode-locking or Q-switching operation.

Some experiments were also carried out on active temporal modulation by Liu et al. and Li et al. In 2013, Z. Liu et al. prepared an all-optical modulator with graphene-coated microfiber (GCMF), using MgF2 as the substrate [119]. When strong light interacts with graphene and pump light intensity varies from 0 to 2.2 W, the absorption of CW probe light in the GCMF will decrease due to the effect of saturable absorption. The high-speed, broadband, all-optical modulation was successfully constructed, and the maximum modulation depth is 5 dB (single-layer graphene) and 13 dB (double-layer graphene), respectively. The modulation bandwidth and speed were recorded at 50 nm and 1 MHz, respectively. The method was simple and effective due to the strong evanescent field of microfiber.

**Figure 9:**
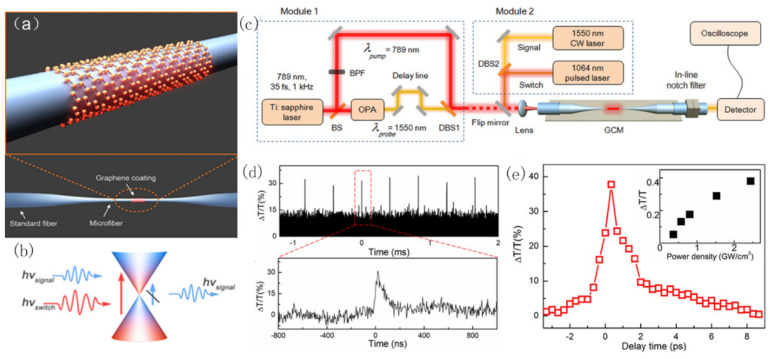
All-optical modulator based on saturable absorption modulation. (**a**) The schematic diagram of a tapered microfiber that is wrapped by graphene. (**b**) The interaction mechanism between light and graphene. Strong switch light stops the absorption of signal light by graphene, leading to intensity modulation of signal light. (**c**) Experimental setup. Module 1: pump-probe setup for measuring the response time of graphene. Module 2: all-optical modulation setup. (**d**) Temporal waveforms of modulated signal light. (**e**) The measured differential transmittance by pump-probe, showing the response time of ~2.2 ps. Reprinted with permission from Ref. [28]. 2014, ACS Publications.

In 2014, Wei Li et al. reported a graphene-clad microfiber all-optical modulator that was able to achieve a modulation depth of 38% and a response time of ∼2.2 ps [28]. As shown in Figure 9a, few-layer graphene is wrapped around a single-mode microfiber to act as a cladding layer. Taking advantage of the mature platform of fiber optics, an infrared signal is coupled into the GCMF and then experiences significant attenuation due to absorption in graphene as it propagates along. When a switch light is introduced, it excites carriers in the graphene. With the Pauli blocking of inter-band transitions, it shifts the absorption threshold of graphene to a higher frequency, resulting in a much lower attenuation of the signal wave. The switch light leads to modulation of the signal output, and its response time is limited by the relaxation of the excited carriers. The GCMF structure enables significant enhancement of the light–graphene interaction via a tightly confined evanescent field guided along the surface of the microfiber.

### 3.2. Kerr Effect

Optical Kerr effect describes the change of refractive index in a nonlinear medium, in which the change of refractive index is proportional to the incident light intensity. The relationship between light intensity and change of refractive index is as explained in equation below:

(4) $$\Delta n=n_{2}I\;$$

where $n_{2}$ is the nonlinear refractive index and *I* is the intensity of the incident light. This optical Kerr effect will give rise to four-wave mixing (FWM), self-phase modulation (SPM), and cross phase modulation (XPM). Similarly, the phase change induced by the change of the refractive index can be expressed as:

(5) $$\Delta \varphi =\frac{2\pi }{\lambda }\Delta nl=\frac{2\pi }{\lambda }ln_{2}I$$

In this case, optical Kerr effect can be used to realize all-optical modulation. 2D materials that had been discussed in Section 2 above exhibit strong third-order nonlinear optical responses with a broad bandwidth, an ultrafast response, and a miniature size. Nonlinear refractive index in graphene was measured in between 10^−11^ to 10^−15^ m^2^/W [102,120,121,122] by numerous researchers while TMD, TI and BP were also reported to show high nonlinear refractive index [5,6,7,8]. We will address each of the nonlinear phenomena: FWM, SPM and XPM and give examples on the types of optical modulator related to it.

**Figure 10:**
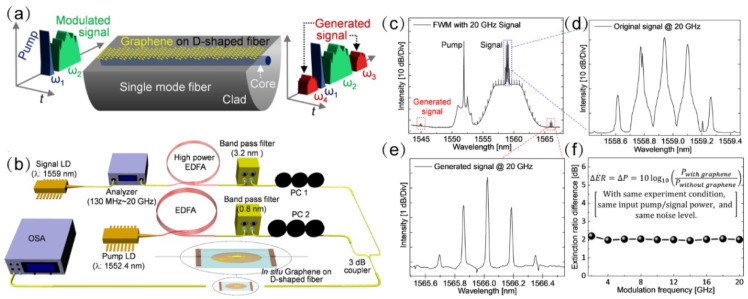
All-optical modulator based on nonlinear four-wave mixing. (**a**) Principle diagram of FWM process by a graphene-coated D-shaped fiber, showing two newly generated signals ($\omega_{3\;}$and $\omega_{4}$) arising from the FWM effect. (**b**) Experiment setup. (**c**) Measured FWM spectrum under 20 GHz input signal. (**d**) Close-up view of the original signal with a modulation frequency of 20 GHz. (**e**) Close-up view of generated signal exhibiting a modulation frequency of 20 GHz (same as the original signal). (**f**) Extinction ratio difference (ΔER) at different modulation frequency. Reprinted with permission from Ref. [131]. 2018, American Chemical Society.

FWM is the wave mixing process, whereby two input waves mix under the phase-matching conditions in a nonlinear medium to produce a new wave [29,123,124,125]. A total of two conditions are required to induce FWM phenomenon; conservation of energy, and phase matching conditions, which is a form of conservation of momentum [126,127]. Figure 10a below shows that when two signals at $\omega_{1\;}$and $\omega_{2\;}$interact with each other in graphene D-shaped fiber, the result is the generation of signals at $\omega_{3\;}$and $\omega_{4}$. The frequencies of $\omega_{3\;}$and $\omega_{4}\;$are determined by conservation of energy and momentum. FWM is commonly employed for wavelength conversion, optical parametric amplification, supercontinuum generation, frequency comb generation, and many other signal manipulations. Experiments showed that the nonlinear FWM effect of 2D materials such as graphene [128,129] and black phosphorus [32,130] are responsible for frequency modulation.

In 2017, Pulak et al. and his colleagues at the Korea University of Science and Technology studied ultra-fast all-optical switches near 1550 nm [131]. The work published can be referred to in Figure 10a–f. A total of two signals from CW laser sources with 1552.4 nm and 1559 nm, respectively, were injected into in-situ growth graphene on a side-polished fiber. The two signals from CW lasers interacted in graphene D-shaped fiber to induce FWM. This subsequently generated signals at wavelengths 1566 nm and 1545 nm. They repeated the experiment with a bare side polished fiber and an improvement of peak intensity at the newly generated signal was observed. This affirmed that the nonlinear response in graphene is significant. They further confirmed the experiment by injecting a modulated signal and the input-modulated signal was copied to the new generated signals.

In 2017, J. Zheng et al. observed frequency modulation using a BP-coated microfiber. A total of two signals were fixed at 1550.70 nm and 1552.35 nm and were amplified first before being launched into the microfiber [31]. This resulted in the generation of two newly converted signals at 1549.05 nm and 1554.0 nm, respectively. Similar to Pulak et al. Zheng also verified that the FWM effect was mainly contributed by BP-coated microfiber [131]. His findings revealed that 74.6% of FWMW observed was from BP. Besides BP, FWM was also demonstrated in MXene and TI materials. Song et al. demonstrated FWM in tapered fiber coated with MXene with conversion efficiency as high as 63 dB at pump power of 140 mW [33]. Signal at 10 GHz was successfully converted to two sidebands. He also measured the nonlinear refractive index of MXene, which was described at −7.5 × 10^−20^ m^2^W^−1^ (800 nm) and −1.22 × 10^−19^ m^2^W^−1^ (15,500 nm). FWM in TI was also proven by Chen et al. [132]. A total of two laser wavelengths at 1541.56 nm and 1545.56 nm, respectively, were injected into a TI tapered fiber, resulting in the generation of two new wavelengths. They experimentally discovered that the wavelength spacing, optical power and polarization states of incident light play an important role in wavelength conversion efficiency. The effective nonlinear coefficient of fabricated TI microfiber was estimated at 1.04 × 10^4^ W^−1^km^−1^ compared to bare tapered fiber 4.059 W^−1^km^−1^. They also commented on the relationship of FWM and the layers of materials. According to Li, FWM intensity increased with the layers numbers when the layers are smaller than 13. When the number of layers is more than 13, FWM intensity decreased exponentially. This is attributed to the higher reflection of incident light and higher reabsorption of FWM in a thick sample.

**Figure 11:**
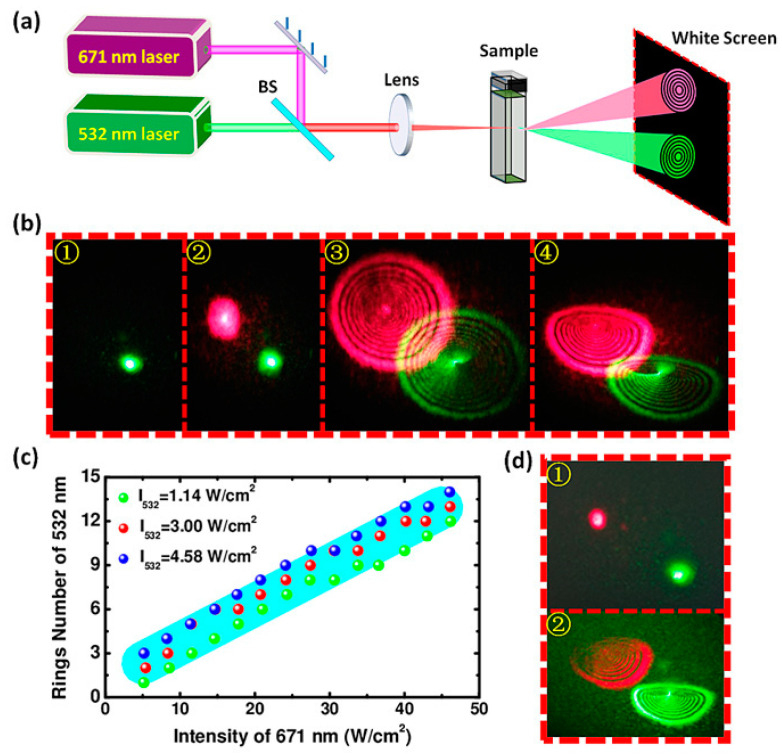
All-optical modulator based on spatial cross-phase modulation. (**a**) Schematic diagram of the experimental set-up. A total of two laser beams at 532 nm and 671 nm are utilized to be signal and pump light, respectively, and focused into the sample by a lens. The diffraction patterns of these two beams are captured on a screen by cameras. (**b**) Different evolution stages of diffraction rings during all-optical modulation. 1: Only weak probe light. 2: Strong pump light starts to interact with sample. 3: Diffraction rings excited by pump light. 4: Diffraction rings collapse from circular to semicircular rings. (**c**) Ring numbers of probe light as a function of pump intensity. (**d**) Diffraction patterns when the 532 nm laser serves as pump light to modulate the 671 nm laser. 1: Two beams start to interact with sample. 2: Final stable diffraction rings [134]. Copyright 2018, WILEY-VCH.

On the other hand, self-phase modulation (SPM) is the phase shift caused by the light field itself when the light wave propagates in the optical fiber, while cross-phase modulation (XPM) is the phase shift caused by the co-propagation light in the same optical fiber. In 2011, R. Wu et al. reported for the first time the spatial self-phase modulation (SSPM) of graphene and elaborated the purely coherent nonlinear optical response of graphene [133]. Following the discovery of nonlinear graphene using SSPM, a large number of investigations into nonlinear optics have extended to other 2D materials, including MoS_2_, SnS and so on. Comparing with the above studies (MoS_2_, 1.8 eV and SnS 1.3 eV), small narrow bandgaps materials such as MXene (0.1 eV) are more suitable to achieve better modulation performance for all-optical modulation. In 2018, Wu at al. demonstrated optical modulation using a spatial cross phase modulation method [134]. A strong pump light at wavelength 671 nm was used to modulate another relatively weak probe light. The variation of intensity of the pump light interacted with Ti_3_C_2_T_x_ MXene dispersion and caused a nonlinear phase change of the probe light. The experimental setup and results obtained for this light-control-light modulator based on spatial cross phase modulation is shown in Figure 11. The intensity of the injected pump light was used to modulate the number of rings formed and the diffraction pattern of the probe light. Similar work was also validated by Lu et al. in 2017 using the same method [135]. A total of two incident laser beams at 532 nm and 633 nm were injected into a few-layer Bismuthene sample. Additionally, Lu and his colleagues also proved that the modulation depth increased as either one of the incident laser beams was increased.

### 3.3. Thermo-Optic Effect

The change of refractive index of a material can also be affected by temperature. This phenomenon is regarded as the thermo-optic effect and is widely used in silicon photonics circuits. The large thermo-optic coefficient of silicon material allows the realization of energy efficient silicon photonic devices with enhanced tunability. The change of refractive index of a material in relation to temperature can be expressed by:

(6) $$n\left(T\right)=n_{0}\left(T_{0}\right)+\frac{dn}{dT}\Delta T$$

where, $n_{0}$ is the refractive index at temperature *T*_0_, *dn*/*dT* is the thermo-optic coefficient and Δ*T* is the change rate of temperature. The change of light phase caused by temperature change is denoted by:

(7) $$\Delta \varphi =\frac{2\pi }{\lambda }\Delta nl=\frac{2\pi }{\lambda }l\frac{dn}{dT}\Delta T$$

**Figure 12:**
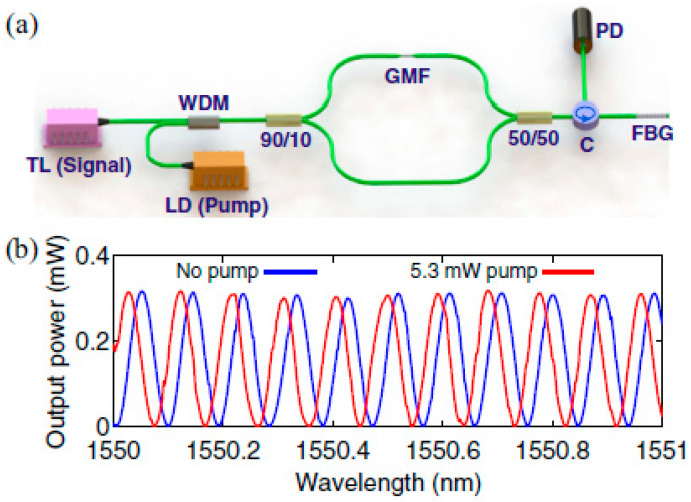
All-optical phase modulation based on graphene microfiber. (**a**) Schematic of the experimental setup for measuring the phase shift in GMF, (**b**) Measured interference fringes with and without pump [110]. Copyright 2017, The Optical Society.

In 2015, Gan et al. reported an all-optical graphene modulator based on phase shifting in a Mach–Zehnder interferometer (MZI) utilizing graphene coated microfiber [136]. Figure 12 below depicts the experimental setup and the observed spectrum shifting. Signal light from 1550 to 1551 nm was multiplexed together with 980 nm pump, which was turned on and off. They observed that the output interference fringe was blue shifted with the increment of pump power. A maximum phase shift of 21π was recorded when the pump power was increased to 230 mW and this translates to efficiency of 0.091 π/mW. From the calculation performed, 230 mW of pump power was equivalent to a temperature rise of 95 K. The microfiber was heated up accordingly by absorbing the heat in the evanescent field. The rise of temperature in graphene tapered fiber changed the refractive index of graphene coated fiber due to the thermo optic effect and subsequently resulted in a phase shift at the output fringe. The experiment was repeated with a bare tapered fiber and the phase shift was verified at 0.18 π under the same experimental conditions. The shifting in bare microfiber was very weak compared to graphene coated microfiber, and it was confirmed that the phase shift induced was contributed by graphene. In another work conducted by Wang et al., he integrated graphene on a microfiber resonator (MFR), which was formed into a self-coupling loop [137]. The interaction between graphene and the microfiber evanescent field allows an efficient generation of ohmic heating, which successively raised the microfiber’s temperature and changed its refractive index. They observed the graphene MFR resonance was red shifted with a linear slope of 71 pm/mW by using a 1540 nm pump. Shifting was observed when the pump power was as low as 3 mW. Wavelength shifting in a bare MFR was just 3.9 pm/mW.

**Figure 13:**
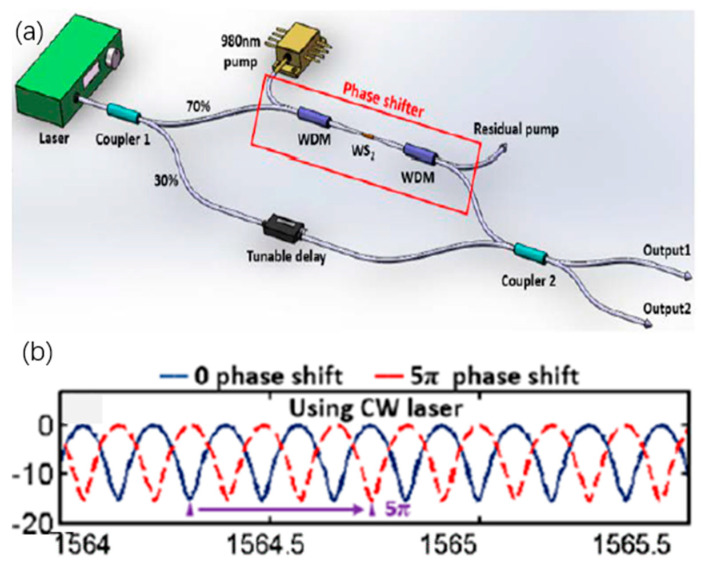
All-optical phase modulation based on WS_2_ microfiber. (**a**) Schematic of the experimental setup of phase shifter based on WS_2_ microfiber fiber, (**b**) Recorded typical spectrum with phase shifting at 5 π. Reprinted with permission from Ref. [139]. 2017, The Optical Society.

Apart from graphene, other 2D materials are also actively under investigation as phase shifters. In 2017, K. Wu et al. reported an all-optical phase shift and switch using WS_2_ deposited on tapered fiber [110]. Unlike graphene, most of the 2D materials exhibit wavelength-dependent absorption due to the presence of band gaps. By selecting the appropriate pump wavelength (control light) in the high absorption region and appropriate signal wavelength in the low absorption region, a thermal optical device can be constructed with high efficiency [48]. Figure 13a shows a typical tungsten disulfide (WS_2_) all-optical phase shifter. The band gap of WS_2_ near 1.3 eV (954 nm) allows the pump (control light) to absorb well at 980 nm, while the signal absorption at 1550 nm is weak [29]. When the control light is irradiated at 980 nm, it is absorbed by WS_2_ and generates heat. Due to the thermo-optical effect, the refractive index of WS_2_ changed with the temperature. Therefore, the 1550 nm signal light transmitted through the tapered fiber deposited by WS_2_ experienced phase shift of 6.1π (0.53 nm to the right), which corresponds to an efficiency of 0.0174 π/mW. Figure 13b illustrates the typical phase shifting at 5π. They also concluded that the thermo-optic coefficient for WS_2_ is a high as 2.88–5.48 × 10^−4^/°C while for silica, it is around 1.1 × 10^−5^/°C. In addition, Wang et al. had demonstrated an all-optical modulator application with phosphorene material using the Mach–Zehnder interferometer to function as a phase modulator [30]. Taking advantage of the physical property of strong light–matter interaction and thickness dependent direct energy bandgap of few-layer phosphorene, a beam signal light can be manipulated by another controlling light. Under the condition of controlling light of 290 mW, the maximum phase shift obtained is 8π with an efficiency of 0.029 π/W under 290 mW pumping. Lately, another work was published on phase shifter using MoWS_2_-rGO coated tapered fiber with phase sift of 0.91 π and efficiency 0.0175 nm/mW [138].

**Figure 14:**
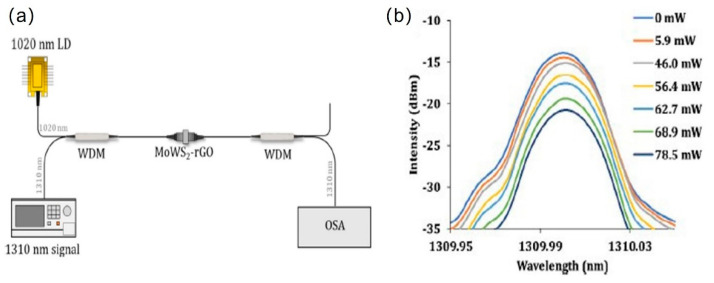
All-optical amplitude modulation based on MoWS_2_-rGO/PVA thin film. (**a**) Schematic of the experimental setup of amplitude modulator based on MoWS_2_-rGO/PVA thin film, (**b**) Amplitude modulation in the band region with the increment of pump power. Reprinted with permission from Ref. [140]. 2019, Elsevier.

On another note, thermo-optics effect is also responsible for amplitude modulation and modulation of light polarization. Amplitude modulation had been proven in both ring and linear fiber cavity, employing TMD and graphene materials. Ahmad et al. constructed their fiber cavity in a ring structure using MoWS_2_-rGO film on a fiber ferrule [140]. Figure 14 shows the proposed linear cavity amplitude modulator and the examined output. When the pump power was increased, the measured peak intensity decreased at three operating regions: O band (1310 nm), C band (1550 nm) and L band (1600 nm). The best recorded amplitude modulation efficiency was 0.17 dB/mW at O band wavelength and its peak reduced from −14 dBm (pump power = 0 mW) to −21 dBm (pump power = 78.5 mW). Later, Ahmad reported another work on amplitude modulator using WS_2_-Chitosan, in a linear configuration [68]. Signal light with center wavelength of 1549.75 nm and pump signal at 980 nm were injected into WS_2_-Chitosan fiber ferrule. Significant decrease in signal peak power was observed when the pump power was raised from 0 nm to 130.1 mW. The attained amplitude modulation efficiency was 0.10 dB/mW. When operating under low light intensity, 2D material will firstly absorb the incident light and enter the saturation state, which is quickly followed by the recovery process. In contrast, if the incident light is of a high intensity, the 2D material may not be able to enter the recovery state and therefore stays in the saturation cycle. If the saturation cycle is still maintained with further increment of light intensity, the thermo-optics effect will be initiated. The high light intensity generates heat, and it further changes the refractive index of the 2D material as well as its supporting structure. This will create the formation of temporal sub bandgap energy, which enhances the absorption of incident light within the 2D material and finally modulates the light intensity of the incident signal.

**Figure 15:**
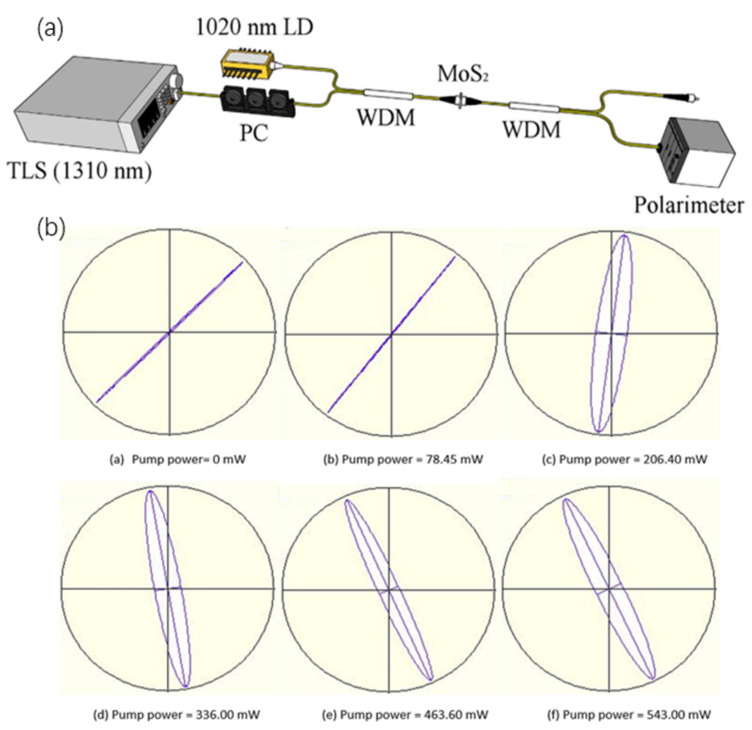
All-optical polarization modulation based on MoS_2_/PVA thin film. (**a**) Schematic of the experimental setup of amplitude modulator based on MoS_2_ PVA thin film, (**b**) Change of light polarization with the increment of pump power. Reprinted with permission from Ref. [142]. 2019, Elsevier.

On the other hand, for polarization modulation, it was firstly demonstrated by Wang et al. by applying polarization interference in control light of 980 nm and signal light with 1550 nm using MoS_2_-PVA in a thin film [141]. Similar work was carried out by Ahmad et al. using MoS_2_ thin film [142]. Figure 15 describes the experimental setup of the polarization modulator. A 1020 nm pump laser diode and 1310 nm band tunable laser source (TLS) were injected into MoS_2_ thin film. Light from this process was further guided into the polarimeter for characterization. The initial light polarization was fixed at 44.73° with an ellipticity of 0.61°. The laser diode pump power was gradually increased to 543 mW and this resulted in modulation of light polarization on the 1310 nm. The light polarization was observed to rotate in an anti-clockwise direction, for a total of 70.81° as the pump power was raised from 0 mW to 543 mW. The high intensity light from the laser diode caused heating in the thin film and subsequently changed the refractive index of MoS_2_ thin film.

## 4. Conclusions and Future Perspectives

We reviewed the recent progress in the all-optical devices based on 2D materials by listing some all-optical modulators based on 2D materials such as temporal, amplitude, phase, frequency, and polarization modulation. The discovery and extensive investigation on 2D materials enables the breakthrough of electronic limit in integrated circuits and builds a foundation for next generation photonics technologies that go beyond Moore’s. As predicted by Feynman, “there is room below the bottom”, which gives a direction for mankind to expand the microscopic quantum world, explore the potential of 2D optoelectronic materials, and to realize the excellent performance index of pursuing all-optical devices with high-speed, low power consumption and a wide operation wavelength range [143].

Predictably, all-optical devices are anticipated to enhance the current telecommunication to perform beyond its limit. To date, a broad range of 2D materials are being studied at a fast pace, which includes a variety of available 2D materials, heterostructures, and hybrid systems. This will allow for new discoveries and concepts in optical modulation. Some proposed area revolving around 2D materials that are worth exploring in the future are listed below.

(1) Explore new heterostructures hybrid systems;
(2) Explore new tailoring mechanism between 2D materials with nano-plasmonic;
(3) Explore the new application of optical or magnetically effect;
(4) Explore the physical mechanism of 2D materials anisotropy.

Scientists from optoelectronics’ and materials’ background should work together towards the goal of optical interconnection that is envisioned to replace the copper network. Therefore, it is essential to develop new all-optical technologies by exploiting new functional materials and to understand the physics of active controlling matter at the nanoscale. With the advancement of nanotechnology and continuous development of materials science, it is reasonable to believe that the era of all-optical interconnection will surely come true and benefit mankind even better.

## Figures and Tables

**Table 1:** Characteristics and properties of various parameters for CNT, graphene, TMD, X-enes, MXene and silicon.

1D Material	2D Materials				3D Material
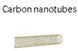	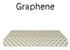	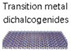	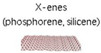	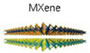	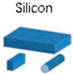
$d_{bcdy}\approx 1\;to\;2\;nm$ $E_{g}\approx 0.4\;to\;0.8\;ev$ $m_{e}\approx 0.1\;m_{o}$ $V_{F}\approx 1\times 10^{8}\;cm/s$ Air stable? Yes	$d_{bcdy}\approx 0.34\;nm$ $E_{g}\approx 0\;ev$ $m_{e}\approx “massless”$ $V_{F}\approx 1\times 10^{8}\;cm/s$ Air stable? Yes	$d_{bcdy}\approx 0.65\;nm$ $E_{g}\approx 1\;to\;2\;ev$ $m_{e}\approx 0.6\;m_{o}$ $V_{F}\approx 5\times 10^{6}\;cm/s$ Air stable? Yes	$d_{bcdy}\approx 0.5\;to\;0.9\;nm$ $E_{g}\approx 0.2\;to\;1.5\;ev$ $m_{e}\approx 0.1\;m_{o}-0.4\;m_{o}$ $V_{F}\approx 5\times 10^{7}\;cm/s$ Air stable? Yes	$d_{bcdy}\approx 1\;to\;2\;nm$ $E_{g}\approx 0.4\;to\;0.8\;ev$ $m_{e}\approx 0.1\;m_{o}-0.3\;m_{o}$ $V_{F}\approx 2\times 10^{7}\;cm/s$ Air stable? Yes	$d_{bcdy}\approx 1\;to\;2\;nm$ $E_{g}\approx 0.4\;to\;0.8\;ev$ $m_{e}\approx 0.1\;m_{o}$ $V_{F}\approx 1\times 10^{8}\;cm/s$ Air stable? Yes
